# Nasogastric Tube Insertion in Intubated Patients: Comparison of Three Different Positions; Standard Sniffing Position, Additional Flexion of the Neck, and Standard Sniffing Position with Lateral Neck Pressure

**DOI:** 10.4274/TJAR.2023.221133

**Published:** 2023-08-18

**Authors:** Shyam Mohanan, Madhu Gupta, Manisha Dabas

**Affiliations:** 1 Department of Anaesthesiology, ESIC Hospital & Pgimsr, Basaidarapur, New Delhi, India

**Keywords:** Anaesthesia, intubation, nasogastric, position

## Abstract

**Objective:**

Our study aimed to evaluate two modified nasogastric tube (NGT) insertion techniques in intubated patients compared to the conventional method in respect of first attempt success rate, time taken for insertion, and complications.

**Methods:**

In this prospective interventional study, patients with orotracheal intubation requiring NGT insertion were randomly allocated into three groups by SNOS Group A (control group- standard sniffing position, n = 40), Group B (additional flexion of the neck, n = 40), Group C (standard sniffing position with lateral neck pressure, n = 40). The number of attempts for successful NGT insertion, time for insertion, and complications were compared.

**Results:**

Modified positions showed a high first-attempt success rate in Group B (55%) and Group C (85%) as compared to conventional Group A (32.50%) (*P* < 0.001). On intergroup analysis of modified groups (B and C), Group C was superior to Group B in 1^st^ attempt success rate with a significant *P* value of 0.003.

**Conclusion:**

In intubated patients, NGT insertion in standard sniffing position with lateral neck pressure has the highest first attempt success rate followed by additional flexion of neck position. Both the modified positions are better positions for NGT insertion in intubated patients.

**Main Points**

• Nasogastric tube (NGT) insertion in intubated patients is many a times difficult to perform due to anatomical reasons as most commonly it gets impacted at the pyriform sinus and arytenoid cartilage.

• Insertion of NGT in an awake state is easier due to the act of deglutition by the patient but in anesthetized and intubated patients, the act of deglutition is not possible.

• Simple manoeuvrers such as additional flexion of the neck and standard sniffing position with lateral neck pressure can overcome the impaction sites and helps in easier and quicker NGT insertion in intubated patients.

• In our study, we found that the standard sniffing position with lateral neck pressure is a superior technique compared to additional neck flexion and standard sniffing position for NGT insertion in anesthetized intubated patients in terms of first-attempt success rate, less time taken, and fewer complications.

## Introduction

The conventional way to insert nasogastric tube (NGT) is often difficult in intubated patients, the failure rate to insert NGT in the first attempt is as high as 50%.^1^ The reasons for failure in inserting NGT in intubated patients are anatomical as well as mechanical. Most of the difficulties are due to anatomical reasons as resistance is felt at the pyriform sinus and arytenoid cartilage while inserting NGT.^2^ Mechanical reasons for difficulty in inserting NGT are due to multiple openings at the distal end of NGT making it prone to kink, coil, and knot,^3^ and the “memory effect” i.e. after a failed attempt followed by subsequent attempts using the same technique, NGT tends to kink again at that location itself^4^ and flexibility of silicone NGT making insertion difficult. When the mechanical reasons add upon the pre-existing anatomical causes further aggravates the difficulty in the insertion of NGT. Simple maneuvers such as additional flexion of the neck,^5^ lateral neck pressure,^4^ and reverse Sellick’s maneuver^3^ were used by several authors for increasing the success rate of NGT insertion. Studies using instruments such as forceps, angiography catheters,^6^ and wire rope^7^ as stylets, and the use of video laryngoscopes such as C-Mac^8^ resulted in success rate, but with complications.

Several authors have reported different techniques with varying success rates. However, we couldn’t find any studies comparing “additional flexion of the neck” and “standard sniffing position with lateral neck pressure” for inserting NGT in intubated patients. The primary objective of this study was to compare 1^st^ attempt success rate between modified positions of inserting NGT (“additional flexion of the neck” and “standard sniffing position with lateral neck pressure”) in comparison to the conventional method. The secondary objective was to find the overall success rate, time taken for insertion, and complications if any.

## Methods

This study was a prospective interventional randomized comparative study conducted after obtaining the permission from Institutional Ethical Committee of ESI-Post Graduate Institute of Medical Sciences and Research and registered in Clinical Trial Registry- India with CTRI registration number CTRI/2020/08/027360. Total of 120 adults of either gender aged between 18 years - 65 years of American Society of Anesthesiologists I and II physical status were included in the study. Exclusion criteria were patients with airway distortion or trauma, neck mass, cervical spine pathology, significant deviated nasal septum, and those taking aspirin or anticoagulants. Informed consent was taken from patients after discussing the study procedure and complications.

The pre-anaesthetic check-up was done in all the patients. A more patent nostril was selected in the pre-operative area based on better fogging on the metal tongue depressor while expiring through each nostril. After giving premedication with midazolam (0.03 mg kg^-1^) and fentanyl (1-2 mcg kg^-1^), induction of anaesthesia was done by propofol (2-2.5 mg kg^-1^) IV and muscle relaxation with vecuronium (0.1 mg kg^-1^) was followed by intubation with appropriate - sized cuffed endotracheal tube. After intubation, oxymetazoline (0.05%) drops were instilled in both nostrils. Sterile, lubricated, 14Fr POLYMED NGT was used.

**Figure 1:**
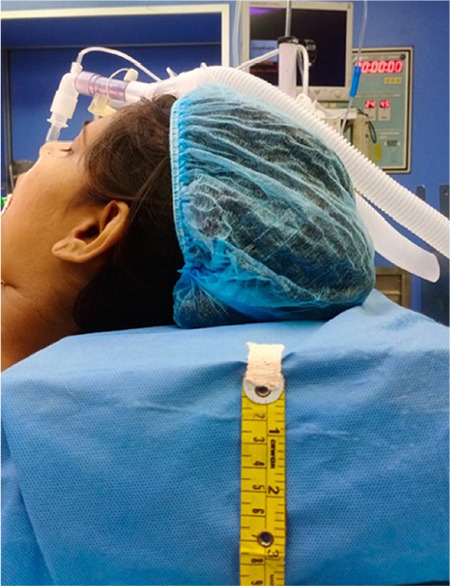
(Group B) NGT insertion in additional flexion of the neck. NGT, nasogastric tube.

**Figure 2:**
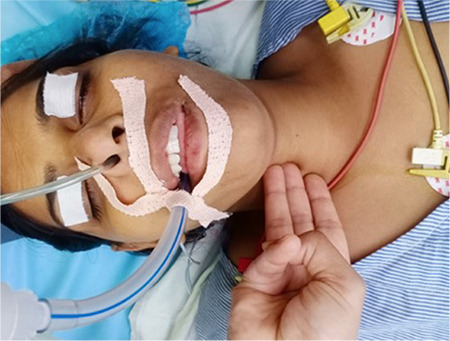
(Group C) NGT insertion in standard sniffing position with lateral neck pressure. NGT, nasogastric tube.

**Figure 3:**
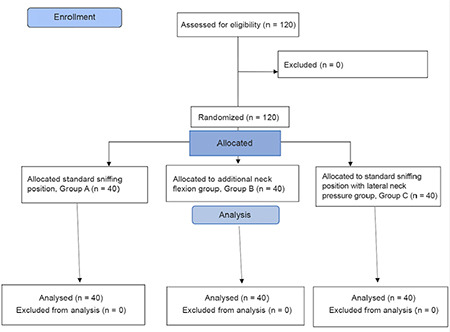
Consortflow diagram of participants in the study group: standard sniffing position group, additional neck flexion group, and standard sniffing position with lateral neck pressure group

In Group A, NGT was inserted while the patient’s head is in the standard sniffing position. In Group B, additional flexion of the neck was used by placing a non-compressible 10 cm width pillow under the patient’s head (Figure 1). In Group C, NGT was inserted while the patient’s head was in the standard sniffing position with applying lateral neck pressure on the same side of the selected nostril (Figure 2). Lateral neck pressure was applied with three fingers placed about an inch lateral to the trachea, at the level of the cricoids cartilage. The successful placement of NGT was confirmed by auscultation at the epigastrium during which a characteristic whooshing sound was heard by injecting 20 mL of air fast into the NGT-called the whoosh test. The time for insertion (seconds) was started from NGT insertion through the selected nostril up to the successful placement of NGT within a maximum of two attempts which includes cleaning and re-lubricating the NGT in case of first attempt failure. If both attempts failed, then the technique was considered a failure, and an alternative technique was used. The following observations were documented: a number of attempts for successful insertion of NGT, time for insertion of NGT, and complications like kinking, coiling, and bleeding (Figure 3).

### Sample Size Calculation

The study by Jonnavithula et al.^5^ observed that the success rate of nasogastric tube insertion in 2 or fewer attempts in the sniffing position was 68% and in the additional flexion position was 92%. Taking these values as a reference, the minimum required sample size with 80% power of the study and 5% level of significance is 40 patients in each study group. So total sample size taken is 120 (40 patients per group).

Analyses were done using IBM SPSS 21.0 (IBM Corp. Released 2012. IBM SPSS Statistics for Windows, Version 21.0. Armonk, NY: IBM Corp. Categorical variables were presented in frequency and percentage (%), and continuous variables were presented as mean ± standard deviation and median (minimum-maximum).

The following statistical tests were applied for the results:

1. The comparison of the variables which were quantitative in nature were analyzed using ANOVA test and post hoc comparison was done using Bonferroni correction for significant parameters.

2. Qualitative variables were compared using the Pearson chi-square test and Fisher-Freeman-Halton test.

For statistical significance, *P* value of less than 0.05 was considered as significant.

## Results

The study was conducted from October 2019 to April 2021. A total of 120 patients were included in the study who had indications for nasogastric tube insertion for elective surgeries. There were no statistically significant differences in terms of age and gender among the three groups (Table 1).

The first-attempt success of NGT insertion in intubated patients was highest in Group C in 34 patients (85%) as compared to Group B and Group A with first-attempt success in 22 patients (55%) and 13 patients (32.5%) respectively (*P* < 0.001). Successful NGT insertion (NGT insertion within 2 attempts) was highest (97.5%) in Group C as compared to Group A (55%) and Group B (80%) (*P* < 0.001). Time taken for successful insertion of NGT (seconds) was less in Group C (23.9 ± 7.5) as compared to Group B (30.09 ± 6.18) and Group A (43.04 ± 12.74) (*P* < 0.001) (Table 2). Intergroup analysis (A vs. B, B vs. C, A vs. C) of the above mentioned procedure parameters were also statistically significant (Table 2).

The occurrence of overall complications was highest in Group A (50%) as compared to fewer complications that occurred in Group B (27.5%) and Group C (7.5%) (*P *< 0.001). Fewer complications were seen in Group C with coiling complication of 7.5% and no complications of kinking and bleeding (Table 3).

## Discussion

This prospective interventional randomized comparative study revealed that modified techniques of NGT insertion, such as additional neck flexion and the standard sniffing position with lateral neck pressure, are effective techniques in inserting NGT in intubated patients in the first attempt with less time and with fewer complications than the conventional method.

The usual sites of resistance while inserting NGT in an intubated patient are seen at the piriform sinuses and the arytenoid cartilages at the same side of the NGT passage.^2^ In an awake state, the upper esophageal sphincter is open during deglutition, thus helping in NGT passage into the esophagus. Inserting NGT after general anaesthesia is difficult because deglutition is impossible, and the sphincter remains closed^9^ and due to compression by the inflated cuff of an endotracheal tube at the esophagus.^10^

There are various studies with high success rate for NGT insertion in intubated patients, which includes slit endotracheal tube assisted,^11,12^ stylet, or guidewire methods,^3,4,6^ using glide scope for placement.^13^ However, these methods have limitations in patients with inadequate mouth opening and are also time-consuming with various complications. Various maneuvers for insertion of NGT such as reverse Sellick’s maneuver,^3,10,14^ neck flexion,^1^ and turning the head to one side^2,15^ have been studied for insertion of NGT.

In our study, additional flexion of the neck (Group B) and standard sniffing position with lateral neck pressure (Group C) were used for NGT insertion in intubated patients. The advantages of these techniques are that the structural changes that occur when the neck is flexed along with the curve of NGT help in the easy passage of NGT into the esophagus by keeping it in the posterior pharyngeal wall and it also prevents glossoptosis.^5^ Lateral neck pressure applied at the same side of NGT insertion compresses the pyriform sinus and medially moves the arytenoid cartilage, allowing NGT to enter the hypopharynx in the usual position^3^ thereby bypassing the anatomical resistance followed by preventing the mechanical reasons for difficult NGT insertion.

Our study revealed that both Group B and C improved the first attempt success rate as compared to Group A by overcoming the anatomical resistance sites. Group C showed highest success rate of NGT insertion within two attempts as compared to Group B and Group A by overcoming the mentioned sites of NGT impaction.

In 2009, Appukutty and Shroff^4^ found that both the head flexion with lateral neck pressure method and slit endotracheal tube method increased the success rate by 82% in the first-attempt insertion of NGT in intubated patients but the bleeding was the persistent complication in the slit endotracheal tube. Observations of Appukutty and Shroff^4^ and that of the present study show that the first attempt success rate of NGT insertion by standard sniffing position with lateral neck pressure happens to be highest with fewer complications. In 2019, Jonnavithula et al.^5^ found higher first-attempt success (76%) in additional neck flexion using a pillow under the head as compared to the standard sniffing position group (63%). Two independent studies by Mandal et al.^3^ and Siddhartha et al.^10^ found good results in neck flexion with lateral neck pressure and reverse Sellick’s group with higher first-attempt success rates of 86% and 77.55% respectively. The present study hasn’t included reverse Sellick’s maneuver to prevent complications during excessive manipulation of the neck.

The time taken for successful NGT insertion in intubated patients was least in the standard sniffing position with lateral neck pressure group (23 ± 7.5 seconds) as compared to the additional neck flexion group (30.09 ± 6.18 seconds) and standard sniffing position group (43.04 ± 12.74 seconds). In 2019, Siddhartha et al.^10^ observed that the time required for NGT insertion was less in reverse Sellick’s group and neck flexion with lateral neck pressure group of 13.05 ± 2.57 seconds and 20.48 ± 4.52 seconds respectively.

In our study, Group C had fewer complications as compared to Group A and B due to the ease of inserting NGT in its usual pathway after the lateral neck pressure. Group C had only coiling complication as compared to coiling, kinking and bleeding complications in Group B and Group A. Thus standard sniffing position with lateral neck pressure technique can be used to avoid unanticipated complications during NGT insertion in anaesthetized intubated patients.

Illias et al.^16^ found that lifting of the thyroid cartilage group and neck flexion with lateral neck pressure group had a lower incidence of complications as compared to the control group. They have emphasized the fact that choosing a procedure with a high success rate that does not require the use of additional instruments can reduce the risk of these complications. In our study, we used simple techniques to avoid all possible complications during NGT insertion.

### Study Limitations

In our study, we avoided obese, pregnant, paediatric, and emergency patients with a full stomach. In the coming years, more studies involving such populations may be required to find the superiority of these modified techniques in such special situations.

## Conclusion

The standard sniffing position with lateral neck pressure technique is the best and most superior technique as compared to the additional neck flexion technique and standard sniffing position for successful and quick NGT placement without instrumentation and hence with less complication. As compared to additional flexion on the neck, the standard sniffing position with lateral neck pressure group is a better technique for successful NGT placement in less time and with fewer complications.

## Figures and Tables

**Table 1:**
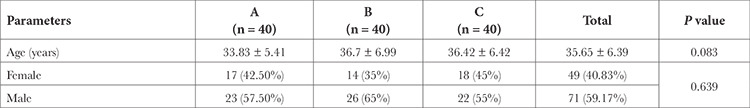
Demographic Parameters

**Table 2:**
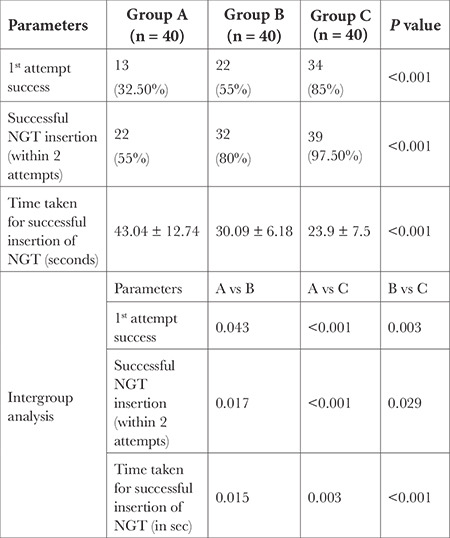
Procedure Parameters

**Table 3:**
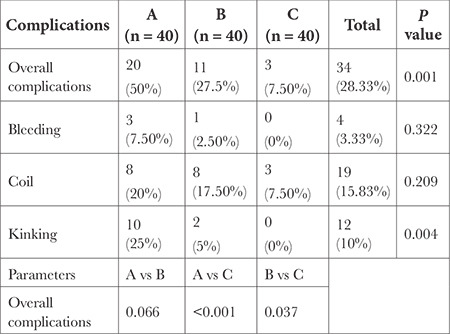
Complications
